# Insecticide Control of *Drosophila suzukii* in Commercial Sweet Cherry Crops under Cladding

**DOI:** 10.3390/insects10070196

**Published:** 2019-07-04

**Authors:** Bethan Shaw, Sebastian Hemer, Madeleine F. L. Cannon, Francesco Rogai, Michelle T. Fountain

**Affiliations:** 1 Pest and Pathology Ecology Department, NIAB EMR, New Road, East Malling, Kent ME19 6BJ, UK; 2 Research and Development Department, Berry Garden Growers Ltd., Tatlingbury Oast, Five Oak Green, Tonbridge, Kent TN12 6RG, UK

**Keywords:** exclusion mesh, integrated pest management, insecticide resistance management, *Prunus avium*, spotted wing drosophila

## Abstract

*Drosophila suzukii* Matsumura is a damaging invasive pest of sweet cherry. Using a series of laboratory leaf contact assays, semi-field, and orchard spray programs we aimed to determine the impact of insecticide programs on *D. suzukii* adult mortality and oviposition in cladding-protected sweet cherry crops. Tests included assessing adult *D. suzukii* mortality after contact with leaves sprayed either one or two weeks previously and emergence of adults from fruits. Spinosad, lambda-cyhalothrin, acetamiprid, lime, pyrethrin, deltamethrin, and cyantraniliprole all reduced fruit damage up to day 7 after application. Of these active ingredients, only spinosad, lambda-cyhalothrin, and cyantraniliprole gave satisfactory control up to 14 days. There was no significant difference in *D. suzukii* mortality when exposed to leaves treated either one or two weeks previously with an application of either spinosad, cyantraniliprole, or lambda-cyhalothrin; however, mortality was significantly higher than *D. suzukii* in contact with untreated leaves. In eight commercial orchards, fortnightly spray applications including spinosad, cyantraniliprole, and lambda-cyhalothrin gave effective control of *D. suzukii* until harvest with very few damaged fruits. These experiments demonstrate that currently approved plant protection products, applied to sweet cherry under protection, give at least two weeks protection from *D. suzukii*.

## 1. Introduction

*Drosophila suzukii* Matsumura was introduced into Europe in 2008 from Asia, probably in imported fruits [1], and first identified in the UK in 2012 [2]. It remains the major pest of soft fruits, including raspberries, blackberries, and strawberries [3,4], and is also a major pest of stone fruits including cherry [5].

Growers employ crop protection practices to reduce the pressure of *D. suzukii* in cropping areas. Insect exclusion mesh is deployed to prevent the incursion of adult flies from the surrounding habitat into the crop [6,7] and, although this has a high initial cost, has been found to be highly effective [8]. The implementation of strict crop hygiene measures including regular fruit picking and the removal of damaged and unmarketable fruit from the cropping area can also significantly reduce fruit damage [9]. Waste fruit removed from the crop needs to be treated to kill any remaining eggs and larvae. Afterwards, the treated waste must be disposed of to prevent re-inoculation of healthy fruits [10]. All of these measures contribute towards control, but fruit growers still apply insecticides because the risk of oviposition into fruits is perceived as too high.

Before the arrival of *D. suzukii*, cherry crops (*Prunus avium* L.) received very few insecticide sprays. An aphicide was normally applied pre-flower to reduce fundatrix of cherry blackfly, *Myzus cerasi* (Fabricius) [11,12]. However, since the arrival of *D. suzukii*, and in order to protect cherries from egg laying it has been necessary to protect fruits, from the white color stage, with insecticides. Before the white stage, fruits are generally not penetrable by the female *D. suzukii* oviscapt and hence insecticides to protect the fruits are not deemed necessary [13]. 

There is a growing trend in Europe, and especially in the UK, to grow cherries under protective cladding. Indeed, over 90% of newly planted cherry orchards in the UK utilize this modern growing system [14]. This ensures high quality fruit by preventing rain damage and subsequent splitting [15,16,17]. This method of cultivation offers two opportunities to help protect cherry fruit from *D. suzukii*. Firstly, there is a barrier to the top of the crop from *D. suzukii* from the plastic cladding potentially reducing immigration from above the canopy. Further, the edges of the crop can be protected by employing insect exclusion mesh [18]. Secondly, the cladding material prevents rain wash-off of applied insecticides, potentially increasing the longevity of the effectiveness of plant protection products compared to open crops [19]. 

Several classes of insecticide provide acceptable control of *D. suzukii* in laboratory and field trials. These include organophosphates, spinosyns, and pyrethroids [20,21,22,23,24]. Additional, less effective products providing shorter periods of protection have also been investigated. These latter products can be integrated into a spray program to help manage insecticide resistance [25] and may be better suited to crops under cladding due to the preservation of products. As many growers have a combination of early-, mid-, and/or late-ripening cherry varieties, the cropping season in the UK can be up to 10 weeks. These factors, in addition to an additional 4 weeks of vulnerable ripening stages [26], requires long-term protection from *D. suzukii*. With a limitation on the maximum number of insecticide applications, growers must rely on a program that includes the rotation of short- and long-term persistence products to gain adequate protection against *D. suzukii*. 

The aim of these studies was to determine the efficacy of plant protection product programs and their impact on *D. suzukii* adult mortality and fruit damage in sweet cherry. In a series of semi-field and orchard trials we firstly screened products for longevity and then assessed fortnightly programs of sprays and the resultant fruit damage in protected cherry crops.

## 2. Materials and Methods 

In 2015, the first experiment screened a range of insecticides for the length of time they protected fruit from *D. suzukii* damage. In 2017, a small field trial examined the effects of weekly vs. fortnightly sprays on adult *D. suzukii* survival in contact with sprayed foliage. In the final year, trials on growers’ commercial crops measured the impacts of a fortnightly spray program on fruit damage and survival of adult *D. suzukii* that came into contact with foliage. 

### 2.1. Efficacy and Longevity of Plant Protection Products 

In 2015, an experimental cherry orchard at NIAB EMR (East Malling, Kent, UK) (cvs. Penny with Sweetheart pollinizer trees, RF 181/182), planted in 2008 was used. The trial took place in two rows of the orchard. The planting distance was 2 m between the trees and 4 m between the rows, which were orientated north–south. The trees were 3.5 m tall and had a 2 m wide canopy, pruned to a center leader, with no canopy <0.5 m from the ground. The crop was protected under a metal tunnel structure (‘Greenhouse’ posts and hoop system from Haygrove Ltd., Ledbury, UK) which was clad in standard commercial plastic cladding (Visqueen Luminance THB cladding also from Haygrove Ltd.), without perimeter insect exclusion mesh.

The plant protection products were applied at approved rates at the time, as shown in Table 1. The trial was a randomized block design with 6 replicates of 8 treatments, including an unsprayed control. The plots in each block were arranged end-to-end in a row, and each plot was three trees—one sprayed tree with an unsprayed guard tree either side and there was a guard row between the two treatment rows. Sprays were applied on 9 July 2015 as a single application with a motorized knapsack sprayer (Birchmeier B245 air-assisted Knapsack mist blower, Stetten, Switzerland) set to a fine spray quality (volume of 1000 L ha^−1^), as shown in Table 1. Dose was calculated based on tree planting density (1250 trees ha^−1^); at 1000 L ha^−1^ this was 800 mL of spray per tree.

Twenty cherry fruits were sampled at random, ensuring that fruit was picked from the upper, middle, and lower canopy, from each sprayed tree on each sample day (days after treatment (DAT) 0, 1, 4, 7, 14) (9, 10, 13, 16, and 23, July 2015) and placed into clear ventilated Perspex boxes (20 × 10 × 10 cm) with a mesh lid. The boxes were maintained for three weeks (at ~20 °C, >40% RH (relative humidity) 16 h light/8 h dark) and assessed for adult *D. suzukii* emergence. Towards the end of the trial some trees had fewer than 20 cherries available, in which case all fruit were picked, and data was analyzed using the numbers of *D. suzukii* per fruit. 

### 2.2. Leaf Contact Bioassay with Spray Programs

In 2017, in the same experimental orchard, we tested the longevity of spray program efficacy on cherry leaves. Each plot was three trees (24 m^2^ soil surface plot^−1^, 2.4 L plot^−1^), separated from adjacent plots by a single guard tree (unsprayed). Fortnightly and weekly spray programs were compared to an untreated control, as shown in Table 2. One plot was sprayed every week (weekly treatment), one plot every fortnight (fortnightly treatment), and one plot remained unsprayed (untreated control), as shown in Table 2 and Table 3. Treatments were applied with a Birchmeier B245 air-assisted Knapsack mist blower, as above. Because of approval restrictions on the numbers of products permitted in one season, Gazelle (acetamiprid) and pyrethrin were incorporated into the weekly spray program.

Before each spray application, 20 leaves from each of the 3 plots were collected and placed into deli cups (145 mm, 115 mm diameter; http://www.reptilesupplyco.com). Five leaves were suspended from the lid inside the pot (4 replicates per treatment) that contained moist filter paper and a sugar feeder (5% (*w*/*v*) dextrose in deionized water solution). Because the sprays were applied on a Friday, the laboratory leaf bioassay was set up on a Monday and the last time each treatment was applied to the foliage was 10 or 17 days previously. Five male and five female laboratory-reared *D. suzukii* were introduced into each pot and then the mortality was assessed 48 h after introduction. The treatments were assessed each week. To understand the impact of the sprays on fruit damage, at the end of the trial, the fruits in the center of the trees were collected and placed into clear ventilated Perspex boxes (20 × 10 × 10 cm) with a mesh lid. Boxes were maintained for 3 weeks (at ~20 °C, >40% RH, 16 h light/8 h dark) and assessed for adult *D. suzukii* emergence.

### 2.3. Fortnightly Spray Trials in Commercial Orchards with and without Mesh

Following the laboratory bioassays and semi-field trials, full fields were conducted at commercial cherry orchards to test the fortnightly spray program in 2018. The two farms selected were in the south east of England at locations where *D. suzukii* was known to occur. At farm site 1, there were five orchards with insect exclusion mesh (<0.9 mm gauge) and one without insect mesh. At farm site 2 both orchards were without insect mesh. As is normal practice, the cherry orchards had a mixture of varieties, as shown in Table 4, and were protected with plastic cladding. The insecticides, as shown in Table 5, were approved at the time of the trial. The growers’ tractor-mounted airblast spray equipment was used to apply the treatments. Water spray volumes were 750 L ha^−1^ (farm 1) and 200 L ha^−1^ (farm 2).

Assessments included adult trap catches; one trap placed within each orchard and one outside the perimeter of the orchard. Biobest traps with Dros’Attract (Biobest Group NV, Westerlo, Belgium) as the liquid bait were used. The traps were filtered weekly to remove captured insects and the number of adult *D. suzukii* was recorded.

The incidence of *D. suzukii* damage to the cherry fruits was assessed each week from white fruit (Biologische Bundesanstalt, Bundessortenamt und Chemische Industrie (BBCH) stone fruit growth stage 81). Forty non-damaged (from cracking), well-shaped, marketable cherries were collected from each plot (20 of each variety). Cherries were picked from the central 10 trees in each of the orchards and from the inside of the canopy on the same day that the leaves for the bioassay were collected. 

Collected fruit was incubated for 2 weeks (~22 °C, >40% RH, 16 h light/8 h dark) in Perspex boxes (20 × 10 × 10 cm) with a mesh lid and the numbers of adult *D. suzukii* emerging from fruit counted. 

In addition, from the orchards coded 1 and 7, as shown in Table 4, 25 medium size leaves were sampled weekly. These were divided into groups of five and compared to unsprayed leaves, collected from NIAB EMR. The same 48 h contact mortality test was carried out in deli cups—as above. Five male and five female (age 4–10 days) *D. suzukii* were introduced into each pot. 

### 2.4. Statistical Analysis

Data was analyzed using Generalized Linear Models (GLM) in R version 3.5.2 [27]. For the insecticide longevity trial (2015), the model ‘Treatment + Row + bs (D, df (degrees of freedom) = 4)’ was fit with the Poisson family with a log link function. The response variable was the number of *D. suzukii* emerging from sampled fruits. To allow for variation along the tree rows, a smoothing spline (function bs, R package ‘splines’ [27] R Core Team, Vienna, Austria, 2018) vs. D on 4 df where D = Distance from the end of the row) was included in the model. To allow for trees where 20 cherries were not available, log(#Cherries) was included as an offset. Overdispersion was estimated by dividing the residual deviance by the residual degrees of freedom; models with a dispersion estimate greater than 1.8 (day 0) were refitted with the quasibinomial family, models with a dispersion estimate greater than 10 (day 14) were refit using a negative binomial GLM—nb.glm from the MASS package [28]. Significances of the main factors were tested using Analysis of Deviance. Comparisons of different treatments vs. the control were made using Dunnett’s test from R package ‘multcomp’ [29]. 

For the spray trials in 2017 and 2018, the model ‘Spray Program × Week’ was fit with the binomial family with logit link function. The response variable was the proportion mortality of *D. suzukii* after 48 h (i.e., dead *D. suzukii* 48 h/total *D. suzukii* introduced). The number of *D. suzukii* introduced in each deli cup was included as a weight (total *D. suzukii* introduced). Overdispersion was estimated as above. Adjustments for complete separation were done by penalized regression using the ‘brglm2′ package in R. Means of different spray programs were compared using Tukey’s honest significant difference (HSD) test at the 5% confidence level. Comparisons were made for treatment within date. 

## 3. Results

### 3.1. Efficacy and Longevity of Plant Protection Products 

**Figure 1:**
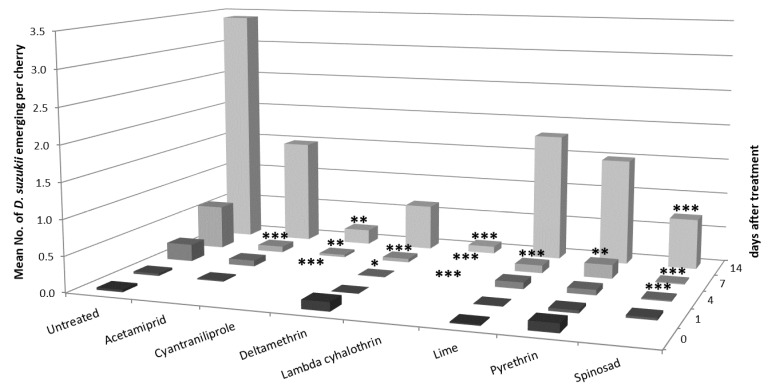
Mean numbers of emerged adult *D. suzukii* from cv. Penny cherries collected 0, 1, 4, 7, and 14 days after spraying in the 2015 trial. Asterisks indicate significant difference to the untreated control using Dunnett’s test (*p* values * <0.05, ** <0.01, *** <0.001) at each time point (*n* = 6) for each individual day. Due to low numbers of *D. suzukii* emerging at days 0 and 1 these were not included in the analyses. For cyantraniliprole (day 4) and lambda cyhalothrin (days 4 and 7) p values could not be calculated with the Generalized Linear Model (GLM) because the emergence of *D. suzukii* from fruit in these treatments was zero.

Natural infestation of *D. suzukii* in monitoring traps and fruit increased over the course of the two-week trial (see untreated control as shown in Figure 1). The numbers of adults emerging from the untreated cherries harvested on days 0 and 1 were too low for statistical analyses, however analyses of emergence from days 4, 7, and 14 were possible, as shown in Figure 1. 

There was a significant difference in the numbers of emerged *D. suzukii* between the rows (day 4: χ^2^(1) = 58.1, *p* < 0.05, day 7: χ^2^(1) = 156.3, *p* < 0.001, day 14: χ^2^(1) = 27.42, *p* < 0.001), along the row (day 4: χ^2^(4) = 441.9, *p* < 0.001, day 7: χ^2^(4) = 889.9, *p* < 0.001, day 14: χ^2^(4) = 156.03, *p* < 0.001) and, importantly, between treatments (day 4: χ^2^(7) = 75.6, *p* < 0.001, day 7: χ^2^(7) = 256.9, *p* < 0.001, day 14: χ^2^(7) = 69.48, *p* < 0.001) on some of the dates. An interaction between row and treatment was only observed for day 14 (χ^2^(7) = 18.6, *p* < 0.01). The efficacy of *D. suzukii* control varied with the plant protection product applied and time post spraying. Spinosad, lambda cyhalothrin, acetamiprid, lime, pyrethrin, deltamethrin, and cyantraniliprole gave good control, significantly reducing emergence in comparison to the control up to day 7 post spraying. Only spinosad, lambda-cyhalothrin, and cyantraniliprole gave control up to 14 days.

### 3.2. Small Scale Leaf Contact Bioassay with Spray Programs

**Figure 2:**
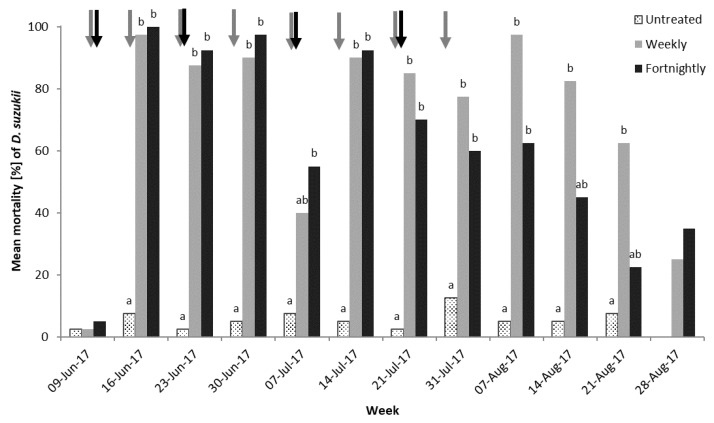
Mean adult mortality (%) of *D. suzukii* 48 h after exposure to cherry leaves sprayed on a weekly or fortnightly plant protection product program compared to an unsprayed control (*n* = 4). The weekly program was; spinosad 9 June, lambda cyhalothrin 16 June, cyantraniliprole 23 June, acetamiprid 30 June, spinosad 7 July, lambda cyhalothrin 14 July, cyantraniliprole 21 July, and pyrethrin 31 July. The fortnightly program was; spinosad 09 June, cyantraniliprole 23 June, spinosad 07 July, and cyantraniliprole 21 July. Data was analyzed using binomial Generalized Linear Models (GLM) with logit link function. Different letters denote significant differences between spray programs within assessment date (Tukey’s honest significant difference (HSD), α = 0.05). Arrows indicate the weekly (grey) and fortnightly (black) spray timings.

In the 2017 leaf bioassay there was no significant difference in mortality of *D. suzukii* in contact with leaves from the untreated, weekly, or fortnightly sprayed trees before the sprays began. In addition, the mortality in the replicated laboratory bioassay with leaves from the untreated control plot was <10% throughout the assay. There was higher mortality when *D. suzukii* made contact with leaves sprayed in the weekly and fortnight programs compared to the untreated control once spray applications began (spray program: Week: χ^2^(22) = 84.3, *p* < 0.001), as shown in Figure 2.

*D. suzukii* adults appeared to have higher mortality from contact with leaves following applications of spinosad, cyantraniliprole, or lambda-cyhalothrin in both the weekly and fortnightly programs, as shown in Figure 2. Following the cessation of sprays, the effects of the insecticides appeared to decline over time (e.g., 7–28 August, Figure 2) and was not significantly different from the control in the final week (Tukey’s test, untreated vs. weekly *p* = 0.955, untreated vs. fortnightly *p* = 0.809, and weekly vs. fortnightly *p* = 1.000). Results were variable depending on the time the assessment was done, post spray application, but, in general either weekly or fortnightly applications of insecticides to cherry foliage gave significantly higher mortality (~90%) compared to untreated leaves (up to 10%), 48 h after exposure. Overall, there was no difference between leaves that had been sprayed either 10 or 17 days previously (untreated vs. weekly *p* < 0.001, untreated vs. fortnightly *p* < 0.001, and weekly vs. fortnightly *p* = 0.523), even after spraying ceased (21 July fortnightly, 31 July weekly sprays), by which time the fruits had been commercially harvested.

The total numbers of *D. suzukii* emerged, per fruit, from the fortnightly, weekly, and unsprayed treatments at the end of the experiment were 0.8, 3.3, and 19.6, respectively (not statistically tested—only one sample).

### 3.3. Fortnightly Spray Trials in Commercial Orchards with and without Mesh

**Figure 3:**
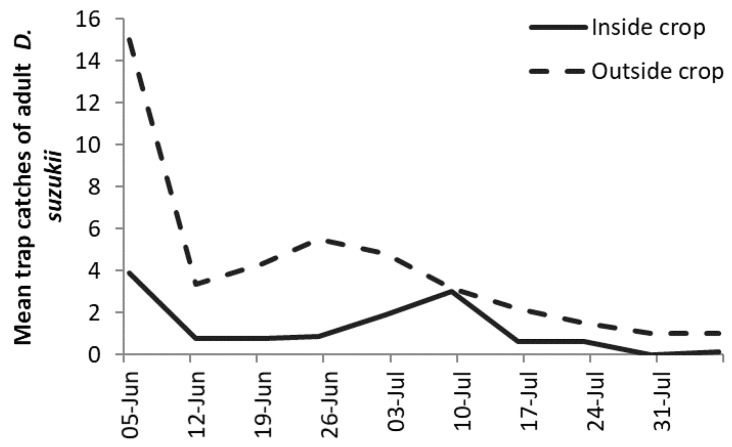
Combined mean trap catches of *D. suzukii* inside and outside exclusion netting during the 2018 field trial.

In the on-farm commercial trials, mean numbers of adult *D. suzukii* captured in the monitoring traps during the fruit ripening period were generally low, as shown in Figure 3. Although numbers at farm site 2 appeared lower than farm site 1, there was no statistical difference in the numbers trapped between the sites (farm site 1 = 6 orchards and farm site 2 = 2 orchards, Tukey’s test, *p* = 0.239). When data was combined for both farm sites there were more *D. suzukii* in the traps around the perimeter of the crop compared to inside the mesh (*p* < 0.001).

From more than 1900 fruits collected during the trial, only one and three *D. suzukii* from farm site 1 and farm site 2, respectively, emerged (data not analyzed).

**Figure 4:**
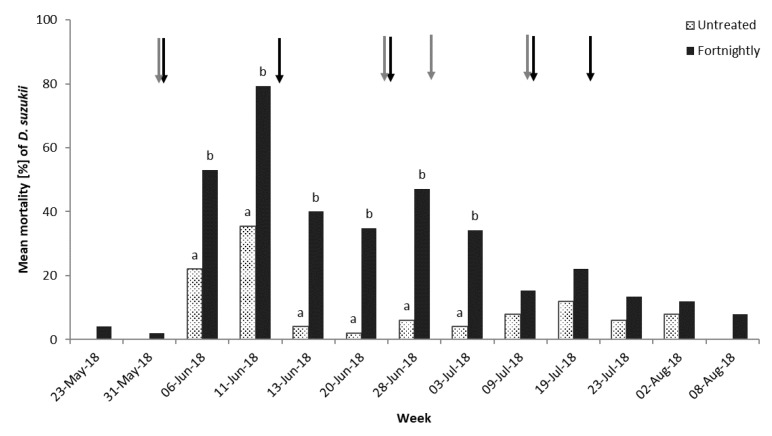
Mean adult *D. suzukii* mortality (%) at 48 h after exposure to cherry leaves sprayed with a fortnightly program compared to an unsprayed control leaves (*n* = 5). Data was analyzed using binomial GLM with logit link function. Different letters denote significant differences at each assessment date (Tukey’s HSD, α = 0.05). Arrows indicate spray applications at farm site 1 (black) and farm site 2 (grey).

Leaves were collected from the commercial trees each week just before the next sprays were applied. Adult flies were introduced to the leaves and mortality assessed after 48 h. There was no difference in mortality between the two sites (χ^2^(2) = 295.06, *p* = 0.084) and data were combined for analyses. The fortnightly spray program resulted in significant *D. suzukii* adult mortality compared to the untreated control (χ^2^(1) = 62.09, *p* < 0.001), as shown in Figure 4. Spraying ceased once harvesting was complete, but until this time there was always higher adult fly mortality compared to the unsprayed cherry leaves. After spraying ceased, adult *D. suzukii* mortality was very similar to contact with unsprayed leaves, as shown in Figure 4.

## 4. Discussion

In these studies, we have investigated the efficacy of plant protection products on *D. suzukii* mortality and fruit damage in cherry crops under protective claddings including how these products can be incorporated into a spray program for season-long control in protected cherries.

In the efficacy and longevity orchard trial there was generally agreement with previous research, concluding that spinosad, lambda cyhalothrin, and cyantraniliprole gave good control; both on adult mortality [22] and fewer *D. suzukii* emerging from fruits [21,30]. The orchard where this trial took place was not a commercial orchard and only the plots in the trial were treated. The pest pressure was high towards the end of the experiment and therefore a robust test of the products.

We firstly screened products for efficacy longevity and encouragingly found several products provided between 7 and 14 days of protection against egg laying. Acetamiprid reduced offspring emergence compared to the untreated control, at 7 days post spraying, although in other studies it is reported as having intermediate effects on mortality when directly applied to adults [31]. The efficacy of acetamiprid on *D. suzukii* adult mortality was shown to be slightly improved by the addition of sugar as a phagostimulant but only when not exposed to rainfall [32]. Hence, cherries under cladding might benefit from a spray bait formulation of acetamiprid but this remains to be tested. The mineral mixture, DsLime, also gave 7 days of fruit protection and is previously reported to reduce oviposition by several researchers [33,34,35,36]. However, there is reportedly limited impact on adult mortality [31,33] and the mechanism is thought to be via an alteration in fruit pH [37]. Lime can leave a chalky residue on fruit [34], but this product is potentially useful in organic systems in combination with other control measures [38] where the numbers of modes of actions of treatments are limited [21,31].

In the on-farm commercial orchard trial, the numbers of *D. suzukii* adults in the monitoring traps from both unmeshed orchards and inside and outside of meshed orchards were relatively low (maximum mean counts in one week was 15 adult *D. suzukii* outside the mesh), but numbers were higher around the perimeter of the orchard. Competition between ripening fruit and traps inside orchards reduces the number of flies detected in monitoring traps, often with trap catches rising only once fruit has been harvested [39].

Spinosad, lambda-cyhalothrin, and cyantraniliprole all significantly reduced the numbers of *D. suzukii* emerging from cherry fruits up to 14 days post foliar application in the first longevity and efficacy trial. Lambda-cyhalothrin, spinosad, and malathion were considered appropriate products toward harvest in US crops leaving low or barely detectable residues in cherry fruit [40]. In both the laboratory and in the field, Shawer et al. [41] found spinosad, lambda-cyhalothrin, and cyantraniliprole not only effective as a preventative method, as in our study, but also as a curative, when applied to cherry fruits after oviposition had occurred. The preventative treatment on cherries resulted in adult mortality of introduced *D. suzukii,* a reduction in the number of eggs laid per female per day, and reduced emergence from fruit. As a curative treatment, when *D. suzukii* inoculated cherries were dipped, these products reduced the hatching rate, development of eggs to larval/pupal stages, and next generation emergence. Within their field trial, Shawer et al. [41] failed to prevent fruit damage with the insecticide programs. However, this was attributed to high pest pressure prior to the start of the program [41]. This stresses the importance of implementing *D. suzukii* control strategies early in the growing season to prevent population growth prior to the appearance of fruit.

Pyrethrin, in our first study, gave up to 7 days protection and significantly reduced *D. suzukii* emergence in comparison to the control. Pavlova et al. [21] were also able to gain significant control using pyrethrin as a preventative treatment but not as a curative treatment, finding no impact on emergence when applied after egg laying. This indicates that growers may benefit from using pyrethrin products early within a spray program when white fruit appears, retaining products such as spinosad and cyantraniliprole, that have both preventative and curative impacts [21,31,41], for later in the program when population pressures are highest.

In the leaf-contact bioassays, the residual impact of protection products on leaves showed that mortality of adult *D. suzukii* exposed to 10 or 17 day old residues varied very little. Weekly or fortnightly spray intervals caused significantly higher mortality than contact with unsprayed cherry leaves when *D. suzukii* adults were exposed for 48 h. Although the mortality assay was done in the laboratory, the leaves were left on the trees for 7 or 14 days post application. Because the crop was under cladding, rainfall could not diminish residues on the leaves [42]. The potential for ultraviolet (UV) degradation [19] and the growth and expansion of leaves and fruit over time could also contribute to the decreasing dose per area over time. As we were still able to identify a similar level of mortality of adults exposed to the leaves between the 7 and 14 day programs, this does not seem to have occurred over the test period of our experiments.

In the commercial farm crops, a 14-day spray regime, which also employed insect exclusion mesh on many of the orchards, saw only four *D. suzukii* emerging from 1900 fruits. Adult trap catches were low inside the cropping area compared to outside indicating that this physical barrier is a positive addition to a longer spray interval management approach [6,7,43]. The initial investment required to deploy insect mesh is high [8], however the long life span of the equipment (expected to be between 7–10 years) results in a physical barrier against *D. suzukii* that can be used for subsequent seasons. This initial cost is also likely to be offset by savings in the use of plant protection products but also labor costs and the handling and disposal of waste fruit. This system has also been highly effective in reducing the immigration of pest numbers including *Halyomorpha halys* Stål (brown marmorated stinkbug), *Cydia pomonella* (Linnaeus) (codling moth), and *Grapholita molesta* (Busck) (oriental fruit moth) into crops [44] from neighboring crops and wild hosts. Therefore, exclusion mesh may also be contributing to integrated pest management (IPM) for other cherry pests. In addition, the structures required to grow cherries or other fruits under cladding can be utilized for the mesh system, removing the need for purpose-built frames and additional costs. *D. suzukii* can move between crops and wild areas throughout the season [6,45] and cherries are one of the first ripening commercial crops in temperate growing regions [46], hence the use of insect mesh can drastically reduce the occurrence of the pest within cropping areas [43].

In both years, for the 14 day spray program, the alternation of spinosad and cyantraniliprole treatments was within (at the time of writing) the maximum number of applications for cherry. Given the emergence of increased insecticide tolerance in *D. suzukii* to spinosad in organic cropping [47], rotation of insecticides with different modes of action to prevent insecticide resistance in conventional systems is crucial [48]. It is essential that products representing different modes of action are effective and long lasting under field conditions whilst the fruit is being harvested. The use of cladding might mitigate insecticide resistance build-up as it may prolong the time post-spraying that products are effective. The extended time of effectiveness would reduce the chances that flies will come into contact with degraded sub-lethal pesticide deposits [24]. This, in combination with other IPM controls, including crop hygiene, exclusion mesh, and tightly timed picking, should reduce and prevent damage caused by *D. suzukii* and avoid resistance developing when the need to use plant protection products is required. It is notable, that post-harvest, and once sprays ceased, the persistence of efficacy reduced over the course of 3 weeks. This is important for resistance management and it is recommended that other non-pesticide controls, e.g., mass trapping, removal of crop debris, and hedgerow management, are employed at this time.

## 5. Conclusions

These experiments demonstrated that plant protection products, currently used in cladding-protected cherry crops in the UK (spinosad, lambda cyhalothrin, cyantraniliprole), give at least 2 weeks protection from *D. suzukii* if rotated in a spray program. Within the manuscript we also highlighted the importance of IPM strategies that can be incorporated into cherry production to not only alleviate the pressure caused by *D. suzukii* in crops but also to reduce resistance build-up to effective plant protection products.

## Figures and Tables

**Table 1:** Plant protection products applied to cv. Penny trees in the efficacy and longevity test in an experimental orchard at NIAB EMR.

Active Ingredient (Ai)	Product Name	Ai L^−1^	Product Rate ha^−1^ (Spray Volume = 1000 L ha^−1^)
Acetamiprid	Gazelle	20% *w*/*w*	375 g
Deltamethrin	Decis	25 g	200 mL
Cyantraniliprole	Exirel	100 g	900 mL
Lambda-cyhalothrin	Hallmark	100 g	0.09 L
Lime(Ca(OH_2_)), Cuprum, ManZincum	DsLime	-	2 kg, 1000 mL, 250 mL
Pyrethrin	Spruzit	4.59 g	12 L
Spinosad	Tracer	480 g	250 mL
Untreated	-	-	-

**Table 2:** Timing of leaf collection and spray program applied to cv. Penny cherry experimental orchard trees and the dates the leaf samples were collected for the laboratory *D. suzukii* adult mortality bioassays. Leaves were sampled from all plots just before the next spray was applied. * Always applied on the weekly program, but only every two weeks in the fortnightly program (NA = not applicable). Leaf sampling continued 4 weeks after the final spray was applied (31 July).

Weekly	Fortnightly	Leaves Collected and then Sprays Applied to Trees *	*D. suzukii* Exposed to Sprayed Leaves (Bioassay Set-Up)
Tracer	Tracer	9 June	14 June
Hallmark	NA	16 June	20 June
Exirel	Exirel	23 June	27 June
Gazelle	NA	30 June	4 July
Tracer	Tracer	7 July	11 July
Hallmark	NA	14 July	18 July
Exirel	Exirel	21 July	24 July
Pyrethrin	NA	31 July	1 August
NA	NA	NA	9 August
NA	NA	NA	15 August
NA	NA	NA	22 August
NA	NA	NA	28 August

**Table 3:** Products and rates of plant protection products used in the cherry leaf bioassay in 2017.

Product	Active Ingredient	Ai L^−1^	Rate ha^−1^
Tracer	Spinosad	480 g	250 mL
Hallmark Zeon	Lambda-cyhalothrin	100 g	90 mL
Exirel	Cyantraniliprole	100 g	900 mL
Gazelle	Acetamiprid	20% *w*/*w*	375 g
Pyrethrum 5EC	Pyrethrin	5% *w*/*v*	4 L

**Table 4:** Cherry varieties in cladding protected commercial orchards used in the farm fortnightly spray program trial in 2018.

Site	Field Code	Insect Mesh (Y/N)	Varieties Assessed
1	1	Y	Kordia, Regina
	2	Y	Merchant
	3	Y	Kordia, Regina
	4	Y	Kordia, Regina
	5	Y	Kordia, Regina
	6	N	Van
2	7	N	Skena
	8	N	Skena, Penny

Y: Yes; N: No.

**Table 5:** Fortnightly spray programs and dates applied by the growers in 2018. At farm site 1 there was also a spray of Calypso (thiacloprid) on 15 April and 2 May, then Batavia (spirotetramat) on 15 May to control aphid and capsid pests; products were applied by the grower according to the label instructions at the time. At farm site 2 there was a spray of Calypso on 13 April for control of capsids, and Batavia on 15 May for aphid control. The rates used were the same as previously described experiments. Hallmark = Lambda-cyhalothrin, Tracer = spinosad, Exirel = cyantraniliprole.

Site 1	Date of Application	Site 2	Date of Application
Hallmark	15 May	Calypso + Tracer	30 May
Tracer	12 June	Exirel	19 June
Exirel	26 June	Tracer	28–30 June
Tracer	10 July	Exirel	7 July
Exirel	24 July		
